# The association between Asian patient race/ethnicity and lower satisfaction scores

**DOI:** 10.1186/s12913-020-05534-6

**Published:** 2020-07-22

**Authors:** Lillian Liao, Sukyung Chung, Jonathan Altamirano, Luis Garcia, Magali Fassiotto, Bonnie Maldonado, Paul Heidenreich, Latha Palaniappan

**Affiliations:** 1 Department of Health Research and Policy, Stanford University School of Medicine, Stanford, USA; 2 Department of Medicine, Stanford University School of Medicine, Stanford, USA; 3 Columbia University Vagelos College of Physicians and Surgeons, 50 Haven Avenue Box #B-26, New York, NY10032 USA; 4 Palo Alto Medical Foundation Research Institute, Palo Alto, USA; 5 Office of Faculty Development and Diversity, Stanford University School of Medicine, Stanford, USA

**Keywords:** Patient satisfaction, Race/ethnicity, Cultural differences

## Abstract

**Background:**

Patient satisfaction is increasingly being used to assess, and financially reward, provider performance. Previous studies suggest that race/ethnicity (R/E) may impact satisfaction, yet few practices adjust for patient R/E. The objective of this study is to examine R/E differences in patient satisfaction ratings and how these differences impact provider rankings.

**Methods:**

Patient satisfaction survey data linked to electronic health records from two large outpatient centers in northern California – a non-profit organization of community-based clinics (Site A) and an academic medical center (Site B) – was collected and analyzed. Participants consisted of adult patients who received outpatient care at Site A from December 2010 to November 2014 and Site B from March 2013 to August 2014, and completed Press-Ganey Medical Practice Survey questionnaires (*N* = 216,392 (Site A) and 30,690 (Site B)). Self-reported non-Hispanic white (NHW), Black, Latino, and Asian patients were studied. For six questions each representing a survey subdomain, favorable ratings were defined as top-box (“very good”) compared to all other categories (“very poor,” “poor,” “fair,” and “good”). Using multivariable logistic regression with provider random effects, we assessed whether the likelihood of giving favorable ratings differed by patient R/E, adjusting for patient age and sex.

**Results:**

Asian, younger and female patients provided less favorable ratings than other R/E, older and male patients. After adjustment, Asian patients were less likely than NHW patients to provide top-box ratings to the overall assessment question “likelihood of recommending this practice to others” (Site A: Asian predicted probability (PP) 0.680, 95% confidence interval (CI): 0.675–0.685 compared to NHW PP 0.820, 95% CI: 0.818–0.822; Site B: Asian PP 0.734, 95% CI: 0.733–0.736 compared to NHW PP 0.859, 95% CI: 0.859–0.859). The effect sizes for Asian R/E were greater than the effect sizes for older age and female sex. An absolute 3% decrease in mean composite score between providers serving different percentages of Asian patients translated to an absolute 40% drop in national ranking.

**Conclusions:**

Patient satisfaction scores may need to be adjusted for patient R/E, particularly for providers caring for high panel percentages of Asian patients.

## Background

Patient satisfaction surveys are increasingly being used by healthcare organizations to monitor patient experience, assess provider performance and inform compensation decisions [1–4]. Similar to other metrics for provider assessment such as cost or clinical quality indicators, patient satisfaction measures may require adjustment for patient characteristics to ensure that reported differences are due to real differences in performance rather than differences in patient mix. Different studies have found patient age, self-perceived health status, education level, sex, marital status, and race/ethnicity (R/E) to be associated with patient satisfaction measures [5–17]. However, which of these associated patient characteristics should actually be adjusted for in practice to ensure valid comparisons among providers is a debate that has yet to reach consensus [18].

The importance of adjusting satisfaction scores is underscored by the fact that the relative ranking of providers is highly sensitive to small differences in mean patient satisfaction scores. Since patient satisfaction data are not normally distributed and left-skewed (the majority of respondents select the highest ratings), a minor difference in mean rating can dramatically lower a provider’s relative ranking [19]. For example, an absolute 3% decrease in mean composite score translates to an absolute 40% drop in national ranking, from 58th percentile to 18th percentile (“A Press, Ganey Report: Medical Practice Means and Ranks,” 2012). Lower scores and ranking can have consequences on a physician’s compensation and mental well-being. In 2013, 2 % of primary care physicians’ compensation and 1 % of specialist physician compensation were based on patient satisfaction metrics [4]. Additionally, several studies have found lower patient satisfaction scores and higher physician burnout to be correlated [20–23], and while the directionality of this relationship is not clear, receiving low scores for reasons beyond one’s control could contribute to burnout.

The Consumer Assessment of Healthcare Providers and Systems (CAHPS) Consortium currently recommends adjusting CAHPS surveys for general health status, age, and education [24]. However, the Medicare Advantage and Prescription Drug Program CAHPS Survey has historically adjusted for additional variables, the most recent of which include mental health status, whether a respondent received help responding, whether a proxy answered questions for the respondent, Medicaid status, low income subsidy, and Chinese language [18]. Furthermore, many studies have advised implementing case-mix adjustment of satisfaction scores and finer reporting of data by additional patient characteristics [10, 12–14, 16, 17, 25, 26]. One such additional patient characteristic that has frequently been studied is patient R/E.

A strong trend that has been noted throughout literature regarding satisfaction scores and patient R/E is that Asian patients provide lower patient satisfaction ratings than non-Hispanic white (NHW) patients [16, 27] and other R/E groups [7, 13, 28–34]. These studies have utilized various survey instruments such CG-CAHPS, which unlike its inpatient counterpart H-CAHPS is not nationally implemented and is only required for accountable care organizations and group practices participating in Medicare and Medicaid initiatives [35]. On the other hand, the Press-Ganey Medical Practice Survey is most commonly used outpatient survey in the U. S [36]. To our knowledge, no largescale studies of Asian patient satisfaction in the outpatient setting using the most prevalent survey instrument have been conducted in the U.S. recently, when patient satisfaction scores are increasingly used to drive physician compensation [4].

To investigate whether patient satisfaction scores should be adjusted for patient R/E, we conducted a large-scale analysis of Press-Ganey Medical Practice Survey data linked to electronic health record data. To assess the generalizability of our findings, we conducted this analysis in two separate outpatient sites in northern California – a non-profit community-based healthcare organization and an academic medical center. We examined R/E differences in patient satisfaction ratings, focusing in particular on Asian patient R/E, and showed how provider scores decline as the percentage of Asians in a provider’s panel increases, an effect that holds even greater consequences when translated to provider ranking. This paper discusses the need to adjust satisfaction scores for Asian patient R/E, especially for providers caring for high proportions of Asian patients.

## Methods

### Setting

#### Site A

Press-Ganey Medical Practice Survey data from December 2010 to November 2014 were obtained from a non-profit organization of community-based outpatient clinics (Site A) serving predominantly urban counties of northern California. Site A serves more than 1 million patients annually whose demographic characteristics reflect the local population with respect to age, sex, and R/E (42.4% White, 23.5% Latino/Hispanic, 6.4% African American, 23.0% Asian) [37]. Providers at Site A are mostly female and have more than 15 years of clinical experience beyond medical school, with a majority of providers being NHW and more than one-third of the providers being Asian [38].

#### Site B

The analysis was also conducted using data from the outpatient sites of an academic medical center (Site B) serving predominantly urban counties of northern California. Press-Ganey Medical Practice Survey data were obtained from March 2013 to August 2014. Site B has over 1500 physicians, oversees approximately 500,000 outpatient visits each year, and like Site A serves a patient population whose demographic characteristics reflect the local population with respect to age, sex, and R/E (42.4% White, 23.5% Latino/Hispanic, 6.4% African American, 23.0% Asian) [37]. Approval of this study was provided by the institutional review boards of both health organizations.

### Patient Satisfaction survey

The Press-Ganey Medical Practice Survey is a commonly used proprietary measure of patient satisfaction [36], and it is composed of 24 core questions grouped into 6 subdomains that assess a patient’s rating of different aspects of care in the outpatient setting: access, moving through the visit, nurse/assistant, care provider, personal issues, and overall assessment. Responses are given using a 1–5 Likert scale, ranging from “very poor” to “very good.”

### Sampling

Five randomly selected patients from each physician were selected each month and mailed surveys, with no more than one survey sent to a patient within 90 days. At Site B, surveys were also available to be completed electronically by patients through individual patient portal accounts (Site A: *N* = 216,293; Site B: *N* = 30,690).

The overall response rates of Site A (17.5%) and Site B (18.0–25.0%) are comparable to other published studies that have used Press-Ganey Medical Practice Survey [39–43]. R/E-specific response rates were available at Site A (NHW: 22.7%; Latino: 14.3%; African-American: 13.6%; Asian: 11.8%; Multiracial/Other Race: 11.9%; Missing/Unknown: 15.0%).

### R/E variable

Patient age, sex, and R/E were extracted from electronic health records and linked to patient satisfaction data. Patient R/E was self-reported. The accuracy of data entry of self-reported patient R/E into electronic health records has been shown to be high, ranging from 92 to 97% [44]. R/E comparisons included NHW, Blacks, Latinos, and Asians. All other racial/ethnic groups (e.g., mixed race, Pacific Islanders) which constitute about 1% of the respondents, and those who did not report their race/ethnicity were grouped into the “Other/Unknown” category.

### Data analysis

#### Score reporting

Press-Ganey survey results are typically reported through three ways: mean score, top-box score, or percentile rank [45]. Because survey responses were left-skewed, violating the linear regression requirement for all variables to be multivariate normal, top-box scoring was used to allow modeling of our outcome as a binary variable using logistic regression analyses. For consistency, we also descriptive and stratified analyses were also reported in terms of top-box scores. In final analyses stratified by panel percentage of Asians, however, mean scores were used in order to match corresponding national rankings provided by Press-Ganey.

#### Descriptive and stratified analyses

Differences in satisfaction scores by patient age, sex, and R/E were examined using chi-square tests. We focused on the care provider subdomain question “likelihood to recommend care provider,” since this question is typically used for physician assessment and compensation [20, 46–49].

#### Regression analyses

For each subdomain, differences in satisfaction scores based on respondent demographic characteristics were examined by performing multivariable logistic regression, adjusting for patient age, sex, and R/E. The outcome of interest was a top-box response (“very good” versus all other categories which included “good”, “fair”, “poor”, and “very poor”). Provider random effects, which use both within- and between-provider variation in the regression estimation, were used to take into account clustering across responses within each provider.

One question was selected from each subdomain to serve as the main assessment question. The questions chosen for analysis in each subdomain were as follows: access (ease of scheduling your appointment); moving through visit (wait time at clinic); nurse/assistant (friendliness/courtesy of the nurse/assistant); care provider (likelihood to recommend care provider); personal issues (our concern for your privacy); overall (likelihood of recommending this practice to others). This main assessment question chosen was that which was most closely associated in meaning with the subdomain overall and had no greater than 10% in missing response. The logistic regression model performed using data from Site A and Site B to assess if consistent R/E differences would be observed.

#### Analysis of provider scores and rankings, stratified by panel percentage of Asians

The impact of increasing percentage of Asian responses on provider scores was assessed by stratifying providers by panel percentages of Asian patients (defined as the proportion of surveys completed by Asian respondents) and comparing mean scores between strata for the subdomain question “likelihood to recommend care provider” [20, 46–49]. For ease of interpretation, the first three strata were cut by intervals of 10% (less than or equal to 10%, 11–20%, and 21–30%) and the fourth and last strata pooled the remaining 31–100% as 10% intervals in this range had very small stratums. In addition to calculating mean scores based on all respondents (regardless of R/E), mean scores based on only NHW and only Asian respondents were calculated. Mean scores were matched to corresponding national rankings provided by Press-Ganey (“A Press, Ganey Report: Medical Practice Means and Ranks,” 2012).

To match mean scores to national rankings provided by Press-Ganey, mean scores according to the scoring method utilized by Press-Ganey were calculated. A provider’s score was defined as an average of the subdomain scores. A subdomain score was an average of all question scores in the subdomain. A question score was given on a 0–100 scale, assigning 0, 25, 50, 75, and 100 to each response category.

Statistical analyses were performed using SAS and STATA. Hypothesis testing was two-tailed and evaluated at the *p* = 0.01 level.

## Results

***Site A.*** Ratings by Asian patients were 16.1 percentage points lower than those provided by NHW patients (*p* <  0.001) (Table 1). Ratings by Latino patients were 2.6 percentage points lower (*p <* 0.001), and ratings by Black patients were 1.4 percentage points lower (*p <* 0.001) relative to NHW patients. Ratings by female patients were 0.6 percentage points lower than those provided by male patients (*p =* 0.001). Relative to patients ages 18–44, ratings by patients ages 45–64 were 7.7 percentage points higher, and ratings by patients 65 and older were 10.3 percentage points higher (*p* <  0.001).

**Table 1:** Demographics characteristics of survey respondents and % top-box response for the subdomain question “likelihood to recommend care provider”

**Demographic Characteristic**	**Non-Profit, Community-Based Clinics (Site A)(*N*** **= 216,392)**			
	**No. (%)**	**% Top-Box**	**∆**	***p*-value**
**Race/Ethnicity**				
Non-Hispanic White (ref.)	134,163 (62.0)	82.4	–	–
Asian	31,377 (14.5)	66.3	−16.1	< 0.001
Black	2705 (1.25)	81.0	−1.4	NS
Latino	13,719 (6.34)	79.8	−2.6	< 0.001
Other/Unknown/Refuse	34,406 (15.9)	76.3	−6.1	–
**Age**				
18–44 (ref.)	45,010 (20.8)	71.7	–	–
45–64	74,872 (34.6)	79.4	+ 7.7	< 0.001
65 or older	96,294 (44.5)	82.0	+ 10.3	< 0.001
**Sex**				
Male (ref.)	76,386 (35.3)	79.3	–	–
Female	140,006 (64.7)	78.7	−0.6	= 0.001
**No. providers**	1186			
**Demographic Characteristic**	**Academic Medical Center (Site B)(*N*** **= 30,690)**			
	**No. (%)**	**% Top-Box**	**∆**	***p*-value**
**Race/Ethnicity**				
Non-Hispanic White (ref.)	21,013 (68.5)	82.9	–	–
Asian	4290 (14.0)	72.9	−10.0	< 0.001
Black	625 (2.0)	77.9	−5.0	= 0.003
Latino	343 (1.1)	81.9	−1.0	NS
Other/Unknown	3983 (13.0)	77.6	−5.3	–
Refuse	436 (1.4)	62.8	−20.1	–
**Age**				
18–44 (ref.)	4488 (14.6)	74.1	–	–
45–64	10,341 (33.7)	81.4	+ 7.3	< 0.001
65 or older	15,861 (51.7)	81.6	+ 7.5	< 0.001
**Sex**				
Male (ref.)	13,616 (44.4)	81.4	–	–
Female	17,071 (55.6)	79.6	−1.8	< 0.001
**No. providers**	2273			

The association between Asian patient R/E and lower top-box rating persisted across all subdomains after adjusting for patient age, sex, and provider random effects (Table 2). In Site A, Asian patients were 11.4 percentage points less likely than NHW patients to provide top-box rating for the overall assessment subdomain. Across subdomains, the difference in likelihood of top-box rating by Asian patients relative to NHW patients was smallest for the subdomain “access” and “care provider” (− 11.4 percentage points), greater for “overall” and “nurse/assistant” (− 14.0 and − 14.1 percentage points), and greatest for “moving” and “personal” (− 14.6 and − 15.3 percentage points). The predicted probabilities for the Asian patient R/E covariate were all significantly different from those for the NHW patient R/E covariate (*p* <  0.001).

**Table 2:** Multivariable analysis results showing the predicted probability (95% CI) of a patient of particular sex, age, or R/E providing a top-box response for each survey subdomain

	Access	Moving Through Visit	Nurse/ Assistant	Care Provider	Personal Issues	Overall
**Site A**						
**NHW (ref)**	0.622 (0.620–0.625)	0.556 (0.554–0.559)	0.781 (0.779–0.784)	0.832 (0.830–0.834)	0.755 (0.753–0.758)	0.820 (0.818–0.822)
**Asian**	0.509^†^(0.503–0.514)	0.410^†^(0.405–0.416)	0.631^†^(0.626–0.636)	0.718^†^(0.713–0.723)	0.602^†^(0.597–0.608)	0.680^†^(0.675–0.685)
**Black**	0.656^†^(0.639–0.673)	0.543 (0.525–0.562)	0.772 (0.757–0.787)	0.818 (0.804–0.832)	0.732 (0.716–0.749)	0.808 (0.793–0.822)
**Latino**	0.627 (0.618–0.635)	0.540 (0.531–0.549)	0.774 (0.767–0.781)	0.817 (0.810–0.823)	0.729^*^(0.721–0.736)	0.808 (0.801–0.814)
**Male (ref)**	0.609 (0.605–0.612)	0.530 (0.526–0.534)	0.753 (0.750–0.756)	0.796 (0.793–0.799)	0.710 (0.707–0.714)	0.786 (0.783–0.789)
**Female**	0.5950^*^(0.593–0.598)	0.524 (0.521–0.527)	0.747 (0.745–0.750)	0.813^†^(0.811–0.815)	0.727 (0.725–0.730)	0.791 (0.789–0.793)
**Age 18–44 (ref)**	0.554 (0.549–0.559)	0.476 (0.471–0.481)	0.714 (0.709–0.718)	0.760 (0.756–0.764)	0.683 (0.678–0.687)	0.739 (0.735–0.743)
**Age 45–64**	0.592^†^(0.589–0.596)	0.552^†^(0.548–0.555)	0.745^†^(0.742–0.749)	0.818^†^(0.815–0.820)	0.725^†^(0.722–0.729)	0.794^†^(0.791–0.797)
**Age > = 65**	0.628^†^(0.625–0.631)	0.530^†^(0.527–0.533)	0.771^†^(0.768–0.774)	0.823^†^(0.820–0.825)	0.739^†^(0.735–0.742)	0.811^†^(0.809–0.814)
**No. responses**	209,289	195,939	206,491	206,883	195,601	207,243
**No. providers**	1184	1182	1185	1185	1184	1184
**Site B**						
**NHW (ref)**	0.648 (0.647–0.648)	0.543 (0.542–0.543)	0.820 (0.819–0.820)	0.862 (0.861–0.863)	0.822 (0.822–0.822)	0.859 (0.859–0.859)
**Asian**	0.522^†^(0.521–0.523)	0.399^†^(0.398–0.399)	0.678^†^(0.677–0.679)	0.759^†^(0.757–0.760)	0.682^†^(0.681–0.683)	0.734^†^(0.733–0.736)
**Black**	0.670 (0.667–0.673)	0.571 (0.569–0.572)	0.791 (0.789–0.793)	0.819 (0.817–0.822)	0.790 (0.789–0.792)	0.831 (0.828–0.833)
**Latino**	0.680 (0.677–0.684)	0.516 (0.513–0.519)	0.758 (0.755–0.761)	0.873 (0.870–0.876)	0.772 (0.770–0.774)	0.859 (0.856–0.862)
**Male (ref)**	0.645 (0.644–0.646)	0.533 (0.532–0.534)	0.811 (0.810–0.812)	0.854 (0.853–0.855)	0.803 (0.802–0.804)	0.847 (0.846–0.848)
**Female**	0.615^*^(0.614–0.616)	0.509 (0.508–0.510)	0.782^†^(0.781–0.783)	0.836 (0.835–0.836)	0.793 (0.792–0.794)	0.831 (0.830–0.832)
**Age 18–44 (ref)**	0.535 (0.533–0.537)	0.450 (0.448–0.452)	0.729 (0.726–0.731)	0.755 (0.754–0.757)	0.746 (0.743–0.748)	0.751 (0.748–0.753)
**Age 45–64**	0.615^†^(0.614–0.616)	0.522^†^(0.521–0.523)	0.791^†^(0.790–0.792)	0.845^†^(0.844–0.846)	0.798^†^(0.797–0.799)	0.840^†^(0.839–0.840)
**Age > = 65**	0.659^†^(0.658–0.659)	0.537^†^(0.536–0.538)	0.811^†^(0.810–0.812)	0.867^†^(0.866–0.867)	0.814^†^(0.813–0.815)	0.861^†^(0.860–0.861)
**No. responses**	24,238	23,751	24,352	24,297	23,868	24,267
**No. providers**	1999	1999	1999	1999	1999	1999

Significantly different from reference group ^*^p < 0.01, ^†^*p* < 0.001

The positive association between age and top-box rating also persisted after adjustment. The association with patient sex was not as strong as those for older age and Asian patient R/E, with few subdomains exhibiting statistically significant differences in predicted probabilities between the two sexes. For the overall assessment subdomain, patient sex was not significantly associated with top-box rating.

***Site B.*** Demographic differences found in Site A were also observed in Site B. Asian patients had the greatest score difference relative to NHW patients, with ratings that were 10.0 percentage points lower than those provided by NHW patients (*p <* 0.001*)* (Table 1). Ratings by Latino patients were 1.0 percentage points lower (*p =* NS), and ratings by Black patients were 5.0 percentage points lower (*p =* 0.003) relative to NHW patients. Ratings by female patients were 1.8 percentage points lower than those provided by male patients (*p <* 0.001). Relative to people ages 18–44, ratings by patients ages 45–64 were 7.3 percentage points higher and ratings by patients ages 65 and older were 7.5 percentage points higher (*p <* 0.001*; p <* 0.001).

As with Site A, the NHW-Asian difference in rating in Site B persisted across all subdomains after adjusting for patient age, sex, and provider random effects (Table 2). In Site B, Asian patients were less likely than NHW patients by 12.5 percentage points to provide top-box rating for the overall assessment subdomain. Furthermore, similar trends in predicted probabilities across subdomains for the Asian patient R/E covariate were also observed in Site B. Across subdomains, the difference in likelihood of top-box rating by Asian patients relative to NHW patients was smallest for the subdomain “care provider” (− 10.3 percentage points), greater for “access” and “overall” (− 12.5 and − 12.6 percentage points), and greatest for “personal,” “nurse” and “moving” (− 14.0, − 14.2, and − 14.4 percentage points). The predicted probabilities for the Asian patient R/E covariate were all significantly different from those for the NHW patient R/E covariate (*p* <  0.001). Similar findings were also observed in Site B with regards to patient age and sex.

**Fig. 1:**
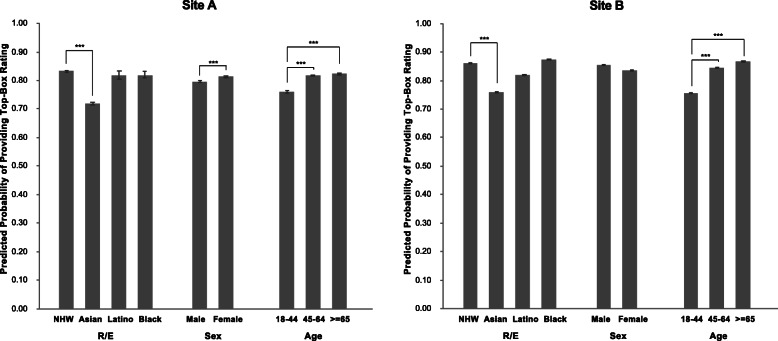
Bar chart visualizing the predicted probability (95% CI) in Site A and Site B of a patient of particular sex, age, or R/E providing a top-box response for the subdomain question “likelihood to recommend care provider.” Significantly different from reference group ^*^p < 0.01, ^***^p < 0.001

Similar trends in predicted probabilities were observed between Site A and B for the care provider subdomain question, which is frequently used for physician assessment and compensation (Fig. 1). In particular, the predicted probabilities of Asian patients providing top-box rating were similar between Site A and Site B (Site A: predicted probability (PP) 0.718, 95% confidence interval (CI): 0.713–0.723; Site B: PP 0.759, 95% CI: 0.757–0.760).

### Analysis of provider Scores and rankings, stratified by panel percentage of Asian patients

**Fig. 2:**
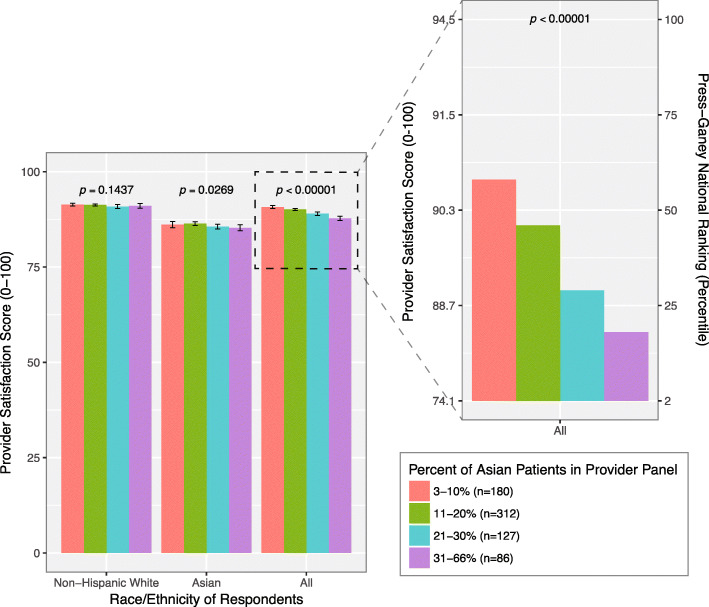
The impact of Asian patient R/E on provider satisfaction score and national ranking. The Press-Ganey mean satisfaction scores (1–100) and respective national rankings (note right-hand side vertical axis of close-up) for providers with varying percentages of Asian patients, considering responses from only Non-Hispanic White patients, and only Asian patients, and all patients regardless of race. Bars indicate 95% CI. Data based on the original site (Site A)

Plotting percentage of Asians in a provider’s panel against provider score for Site A demonstrated that overall provider score declines as the percentage of Asians in a provider’s panel increases (Fig. 2). When we only considering responses provided by either NHW or Asian patients alone, mean score does not significantly change as the percentage of Asians in a provider’s panel increases (*p* = 0.1437 and *p* = 0.0269, respectively). However, when considering responses from all respondents regardless of R/E, the mean score significantly declines as the percentage of Asians in a provider’s panel increases (*p* <  0.00001). Similar trends were observed in Site B.

Collectively considering responses from Asian and NHW patients, an increase in the percentage of Asian patients in a provider’s panel from low (3–10%) to high (> 30%) is associated with a 40% drop in national ranking, from 58th to 18th percentile (Fig. 2**, right-hand side vertical axis of close-up**).

## Discussion

We found that Asian patients, compared to NHW patients, were less likely to give the highest rating on satisfaction surveys, even after adjusting for patient age, patient sex, and provider random effects. This Asian-NHW difference in rating was observed in two different outpatient settings with high densities of Asian patients – a non-profit organization of community-based clinics and an academic medical center located in northern California. The effect of Asian R/E was stronger than effects observed for patient sex or age, two factors for which patient satisfaction scores are commonly adjusted when comparing providers [8, 11, 50–52].

We hypothesize that the observed Asian-NHW rating difference is more likely due to R/E differences in survey response than differential care. It has been consistently observed that Asians are less likely to select extremes and less likely to choose top-box responses to surveys [17, 53–55]. This effect is exaggerated when questions are rated subjectively using Likert-type scales [56–58]. Despite providing lower satisfaction ratings, Asian patients are not more likely to change providers, indicating that lower ratings may not necessarily imply greater dissatisfaction with care [27, 30]. Our hypothesis is strongly supported by a study evaluating patient satisfaction in an objectively quantifiable domain—wait time. Given the same wait time for an office visit, Asian patients were found to perceive longer wait time and be less satisfied with wait time compared to NHW patients [59]. The Asian-NHW rating difference is also observed in other domains such as life satisfaction. Asians express lower levels of overall life satisfaction than NHWs, despite greater educational attainment and income which are known to be correlated with life satisfaction [60]. Together, these findings suggest that the observed lower satisfaction ratings provided by Asian patients may be due to R/E differences in survey response and that scores should be adjusted for Asian patient R/E to prevent inaccurate comparison of satisfaction scores across providers.

We showed that providers with higher percentages of Asian patients have markedly lower rankings than providers with lower percentages of Asian patients (Fig. 2, **close-up**). This is consistent a study showing that lower CAHPS patient experience ratings in California (which has the second highest Asian population in the U.S.) compared to the rest of the nation are primarily driven by lower ratings provided by Asians compared to other R/E groups in California; this rating difference disappears only after adjusting for several variables including race/ethnicity [13]. Whether it be at the provider-level or state-level, the satisfaction scores of provider serving regions with larger Asian populations may be disproportionately affected by the Asian-NHW patient rating difference.

The decision to adjust for R/E when using patient satisfaction as a metric is contingent on whether Asian patients are actually receiving inferior care. If so, adjustment should be avoided to prevent erasing meaningful differences in patient satisfaction. However, given evidence that Asian patients are not receiving inferior care, adjustment can be considered for physicians caring for more than or equal to 10% Asians or when the difference in panel percentage of Asian patients is 10% between the highest and lowest providers. When comparing responses from all patients regardless of R/E with responses from only NHW patients, mean scores are significantly different at an alpha level of 0.05 for panel percentages greater than 3–10% (Supplemental Table 1). However, it is important to remember that adjustment is not an either/or decision; administrators can examine both race-adjusted and unadjusted data to determine how to best utilize results.

## Limitations

A few limitations should be considered when interpreting the results of the study. One limitation is that the region under study has a larger Asian population than other parts of the country, and that the observed Asian-NHW rating difference may not be found in areas with smaller Asian populations. However, if is true that the Asian-NHW rating difference is driven by R/E difference in survey response, and that this rating difference is only observed in areas with larger Asian populations, then accounting for this rating difference should have even greater relevance when using national rankings to compare providers across populations with varying percentages of Asians.

Low response rates of 17.5% and 18.0–25.0% for Sites A and B, respectively, pose a second limitation and could potentially bias our results. However, our “real-world” response rates are comparable to those reported elsewhere and in studies that have used the same Press-Ganey Medical Practice Survey [39–42]. The fact that low response rates are commonly observed for Press-Ganey satisfaction surveys underscores the importance of studying survey response and its effect on providers’ scores. Furthermore, it is important to remember that even if patient satisfaction scores may not necessarily be a “true” measure of provider performance (due to low response rates or other factors), they are considered “true” in the eyes of the hospital and reimbursement systems that currently utilize them. Therefore, it is important to study patient satisfaction scores and base adjustment decisions within the settings in which these patient satisfaction surveys are implemented.

Variation in response rates among R/E groups is also a potential source of bias. Compared to the local population, the survey respondents had a higher percentage of NHWs and lower percentage of Asians, Latino/Hispanics, and African Americans. Despite this information, we cannot make any conclusions about directionality of R/E-specific response biases (i.e. whether the NHWs that responded were more satisfied than the average NHW or whether Asians that responded were less happy than the average Asian). R/E-specific survey response rates have been found to vary depending on survey mode, length of questionnaire, survey language, and cultural sensitivity [61]. More work is needed to understand R/E-specific survey response behaviors and to create surveys that more representatively sample R/E minority groups.

Our inability to assess differences in quality of care is another limitation of the study. We cannot exclude the possibility that Asians were provided lower quality care. However, studies investigating R/E differences in quality of care in terms of clinical measures can provide additional insight as to whether Asians are truly receiving inferior care compared to other R/E groups. Asian patients enrolled in Medicare+Choice have been found to receive equal or better frequency of care compared to NHW patients for mammograms, use of beta-blockers, diabetes care, and testing for cholesterol and blood pressure [62]. However, Asian patients enrolled in traditional Medicare have also been found to be less likely than NHW patients to receive screening and diabetic services in several major metropolitan statistical areas with large Asian populations [63]. In terms of clinical outcomes, Asian patients enrolled in Medicare+Choice have been found to have lower blood pressure, lower cholesterol levels, and similar glycated hemoglobin levels compared to NHW patients even after adjustment for age and sex [64]. Together, these studies suggest that difference in quality of care between Asians and NHWs may depend on various factors, such as health plan and region. More work is needed to define “quality of care” and determine what measures appropriately capture its differences among R/E groups.

Data on Asian subgroups, language spoken, degree of acculturation, and immigration status at both sites were limited, preventing finer examination of the effect of Asian R/E. Primary care experience has been found to vary among Asian subgroups and between native and non-native Asian speakers of English [34, 65] and sampling bias has been shown to promote overrepresentation of English-speaking and well-acculturated Asians in surveys [66]. When surveyed about life satisfaction, U.S.-born Asians are less likely than foreign-born Asians to be “very happy” as opposed to “pretty happy” [60]. It is possible that this observed Asian-NHW rating difference is a result of one or more Asian subgroups with greater language fluency and acculturation masking less represented subgroups. As a result, findings from our study might be generalizable to the Asian population in northern California broadly consisting of the largest Asian subgroups (Chinese, Asian Indian, Vietnamese, Filipino, Korean, Japanese) with higher education, English fluency, and income compared to the Asian population in Minnesota which is drawn narrowly from one of the largest Hmong communities in the U.S. [67] With a majority of studies on this topic being conducted through secondary data analysis, there is a need to improve coding of R/E, linguistic, and acculturation information in health surveys to enable more disaggregated analyses in the future.

## Conclusion

Asian patients were less likely than NHW patients to provide top-box rating for the Press-Ganey Medical Practice Survey, even after adjusting for age, sex, and provider random effects. Existing literature showing that Asians tend to select less extreme responses suggests that the Asian-NHW difference in rating may be due to R/E differences in survey response rather than differential care. Therefore, providers with higher panel percentages of Asian patients may be unduly penalized in their satisfaction scores, an effect that is further exaggerated in the ranking context. When compensation incentives are based on patient satisfaction results and R/E composition varies across providers, scores may need to be adjusted for Asian patient R/E to avoid penalizing providers with higher panel percentages of Asian patients.

## Supplementary information

**Additional file 1: Table S1.** The Press-Ganey mean satisfaction scores (1–100) for providers with varying percentages of Asian patients, considering responses from only Non-Hispanic White patients, and only Asian patients, and all patients regardless of race. These values correspond to those shown in Fig. 2. Data based on the original site (Site A).

## Data Availability

The datasets used and/or analyzed during the current study are available from the corresponding author on reasonable request.

## Abbreviations

R/E: Race/ethnicity
NHW: Non-Hispanic white
CAHPS: Consumer Assessment of Healthcare Providers and Systems
PP: Predicted probability
CI: Confidence interval
