# Supplementary material for: The association between Asian patient race/ethnicity and lower satisfaction scores
Source: BMC Health Serv Res. 2020 Jul 22;20:678. doi: 10.1186/s12913-020-05534-6 (PMC7374891; doi:10.1186/s12913-020-05534-6)
Supplement: Supplementary file 1 — Additional file 1: Table S1. The Press-Ganey mean satisfaction scores (1–100) for providers with varying percentages of Asian patients, considering responses from only Non-Hispanic White patients, and only Asian patients, and all patients regardless of race. These values correspond to those shown in Fig. 2. Data based on the original site (Site A). [file 12913_2020_5534_MOESM1_ESM.docx]

**SUPPLEMENT**

**Supplemental Table 1.** The Press-Ganey mean satisfaction scores (1-100) for providers with varying percentages of Asian patients, considering responses from only Non-Hispanic White patients, and only Asian patients, and all patients regardless of race. These values correspond to those shown in Figure 2. Data based on the original site (Site A).

| **Race/Ethnicity of Respondents** | **Provider Satisfaction Score (95% CI)** |
| --- | --- |
| Non-Hispanic White | 91.37 (90.99-91.75) |
| Non-Hispanic White | 91.27 (90.99-91.55) |
| Non-Hispanic White | 90.87 (90.35-91.39) |
| Non-Hispanic White | 91.05 (90.43-91.67) |
| Asian | 86.14 (85.33-86.95) |
| Asian | 86.38 (85.88-86.88) |
| Asian | 85.61 (84.99-86.24) |
| Asian | 85.29 (84.50-86.07) |
| All | 90.73 (90.34-91.11) |
| All | 90.10 (89.81-90.38) |
| All | 89.00 (88.54-89.46) |
| All | 87.75 (87.20-88.31) |
